# Trends in Outpatient Antibiotic Prescriptions in the United States During the COVID-19 Pandemic in 2020

**DOI:** 10.1001/jamanetworkopen.2021.26114

**Published:** 2021-09-22

**Authors:** Deanna J. Buehrle, Marilyn M. Wagener, M. Hong Nguyen, Cornelius J. Clancy

**Affiliations:** 1 VA Pittsburgh Healthcare System, Pittsburgh, Pennsylvania; 2 Department of Medicine, University of Pittsburgh, Pittsburgh, Pennsylvania

## Abstract

This cross-sectional study examines the prescription fills of commonly prescribed outpatient antibiotics in the US through the end of 2020.

## Introduction

Following the arrival of COVID-19 in the US, there were significant reductions in prescription fills with commonly prescribed outpatient antibiotics in April 2020.^1^ Through July 2020, monthly azithromycin, amoxicillin, amoxicillin-clavulanate, and levofloxacin prescription fills did not rebound significantly.^1^ In this cross-sectional study, we measured prescription fills of commonly prescribed outpatient antibiotics in the US through the end of 2020.

## Methods

This cross-sectional study was found exempt by the institutional review board at the University of Pittsburgh. Informed consent was waived because data was deidentified. This study followed the Strengthening the Reporting of Observational Studies in Epidemiology (STROBE) reporting guideline.

During each quarter, the mean monthly prescription fills for 9 of the 10 most commonly prescribed US outpatient antibiotics (excluding sulfamethoxazole-trimethoprim) was found from IQVIA National Prescription Audit data (Figure).^1^ A nonlinear least squares regression model was used to anticipate mean monthly prescription fills during quarters of 2020 based on 2015 to 2019 data. Anticipated and actual mean monthly fills each quarter were compared by a 2-tailed *t* test. Statistical significance was set at *P* < .05. STATA/SE version 16.1 (StataCorp) was used for statistical analysis.

**Figure.**
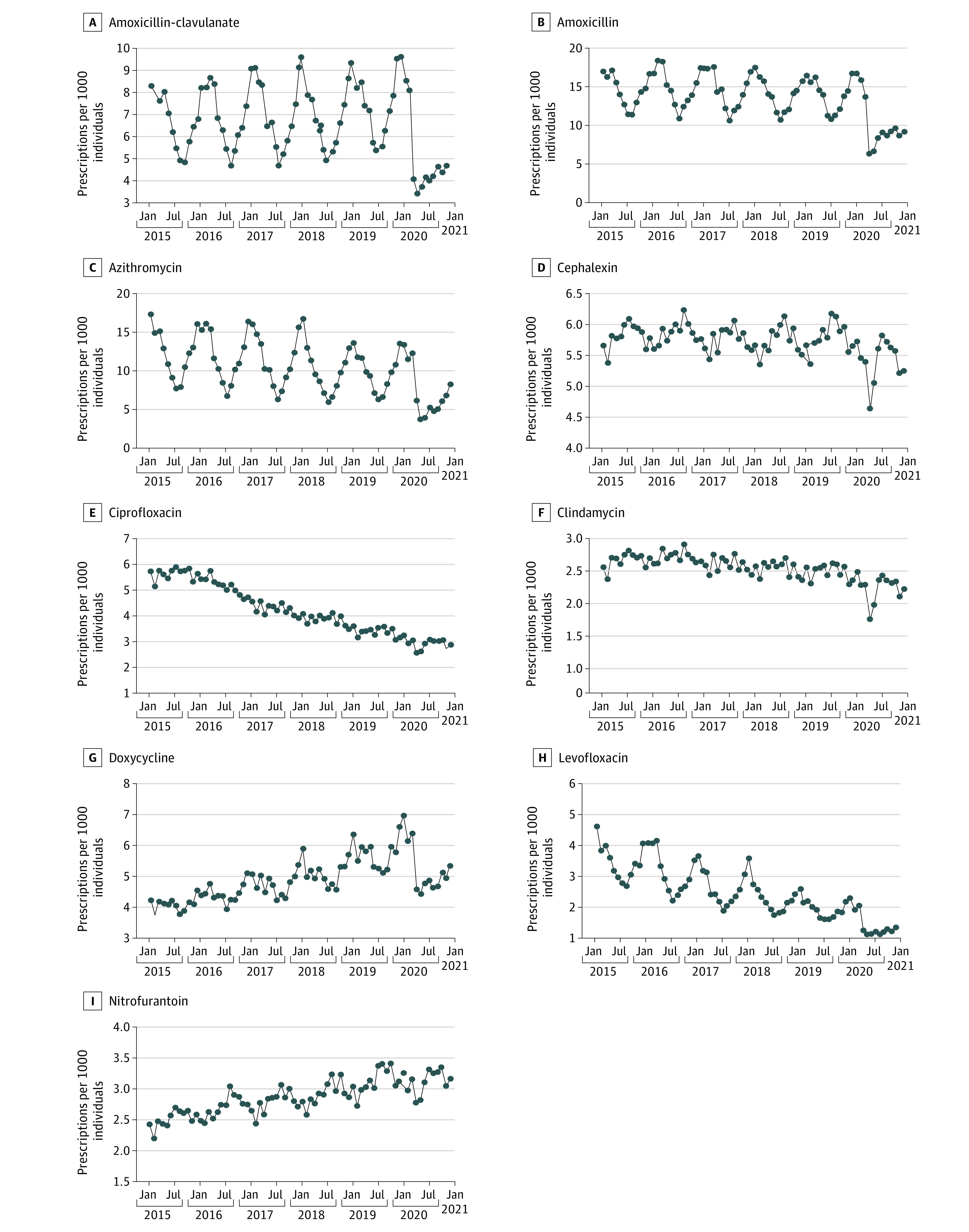
Outpatient Antibiotic Prescription Fills in the United States, January 2015 Through December 2020 Data are for 9 of the 10 most commonly prescribed outpatient antibiotics in the United States, excluding sulfamethoxazole-trimethoprim (for which complete 2020 data were unavailable). The beginning of the COVID-19 pandemic nationally is taken to be March 2020. Monthly prescription fill data are presented as filled black circles. Prescription fill data are from IQVIA National Prescription Audit databases, which project total retail prescription drug sales using information from over 93% of retail pharmacies in the US. The data include prescriptions for adults or children stemming from encounters in any setting, including in-person and telemedicine visits, and from telephone or electronic communications. Population data are taken from US Census monthly estimates.

## Results

In quarter 1 of 2020 (ie, January-March), the mean monthly prescription fills of each agent did not significantly differ from anticipated values (Table). In quarter 2 (ie, April-June), quarter 3 (ie, July-September), and quarter 4 (ie, October-December 2020), the mean monthly amoxicillin, amoxicillin-clavulanate, and doxycycline fills were significantly lower than anticipated (amoxicillin: 25.3%-44.1%; *P* = .003-.03; amoxicillin-clavulanate: 30.1%-40.0%; *P* = .005-.02; doxycycline: 17.8%-20.7%; *P* = .008-.01). Azithromycin fills were significantly lower than anticipated in quarters 2 and quarter 4 (quarter 2: 35.4% decrease; *P* = .02; quarter 4: 31.8% decrease; *P* = .03). The decrease in quarter 3 was not significant (22.1% decrease; *P* = .06).

**Table.** Outpatient Antibiotic Prescription Fills by Quarter in the United States, January 2015-December 2020^a^

Drug	Quarter, 2020			
	January to March	April to June	July to September	October to December
Amoxicillin				
Anticipated monthly mean^b^	16.16	12.59	11.98	14.32
Observed monthly mean^c^	15.35	7.04	8.95	9.11
Difference (95% CI)^d^	–0.81 (–2.12 to 0.49)	–5.55 (–9.98 to –1.12)	–3.03 (–3.73 to –2.33)	–5.20 (–8.89 to –1.51)
Difference (% change)	–5.01	–44.1	–25.3	–36.4
*P* value	.12	.03^e^	.003^e^	.03^e^
Azithromycin				
Anticipated monthly mean^b^	12.33	7.10	6.43	10.32
Observed monthly mean^c^	12.37	4.59	5.01	7.04
Difference (95% CI)^d^	0.04 (–5.06 to 5.13)	–2.51 (–4.07 to –0.95)	–1.42 (–2.99 to 0.14)	–3.28 (–5.59 to –0.97)
Difference (% change)	0.3	–35.4	–22.1	–31.8
*P* value	>.99	.02^e^	.06	.03^e^
Amoxicillin-clavulanate				
Anticipated monthly mean^b^	8.86	6.64	6.37	8.06
Observed monthly mean^c^	8.81	4.03	4.45	4.84
Difference (95% CI)^d^	–0.05 (–0.95 to 0.83)	–2.60 (–3.38 to –1.82)	–1.92 (–2.47 to –1.36)	–3.21 (–5.32 to –1.11)
Difference (% change)	–0.6	–39.3	–30.1	–40
*P* value	.81	.005^e^	.005^e^	.02^e^
Doxycycline				
Anticipated monthly mean^b^	6.17	5.79	5.81	6.24
Observed monthly mean^c^	6.49	4.59	4.73	5.13
Difference (95% CI)^d^	0.33 (–0.44 to 1.10)	–1.19 (–1.71 to –0.67)	–1.08 (–1.53 to –0.68)	–1.11 (–1.36 to –0.45)
Difference (% change)	5.2	–20.7	–18.6	–17.8
*P* value	.20	.01^e^	.001^e^	.01^e^
Cephalexin				
Anticipated monthly mean^b^	5.49	5.94	6.01	5.72
Observed monthly mean^c^	5.44	4.76	5.75	5.14
Difference (95% CI)^d^	–0.06 (–1.26 to 1.14)	–1.18 (–2.91 to 0.55)	–.26 (–0.58 to 0.05)	–0.58 (–1.15 to –0.00)
Difference (% change)	–0.9	–19.9	–4.3	–10.1
*P* value	.85	.10	.07	.05^e^
Nitrofurantoin				
Anticipated monthly mean^b^	3.05	3.26	3.35	3.32
Observed monthly mean^c^	3.13	2.89	3.28	3.18
Difference (95% CI)^d^	0.08 (–0.41 to 0.57)	–0.37 (–0.72 to 0.00)	–0.08 (–0.17 to 0.03)	–0.14 (–0.49 to 0.22)
Difference (% change)	2.6	–11.3	–2.1	–4.2
*P* value	.55	.06	.08	.24
Ciprofloxacin				
Anticipated monthly mean^b^	2.78	2.80	2.71	2.52
Observed monthly mean^c^	3.08	2.70	3.04	2.88
Difference (95% CI)^d^	0.29 (–0.16 to 0.74)	–0.10 (–0.59 to 0.39)	0.33 (0.29 to 0.37)	0.36 (0.06 to 0.67)
Difference (% change)	10.8	–3.6	12.2	14.3
*P* value	.11	.38	.001^e^	.04^e^
Clindamycin				
Anticipated monthly mean^b^	2.40	2.49	2.49	2.38
Observed monthly mean^c^	2.35	2.03	2.37	2.23
Difference (95% CI)^d^	–0.05 (–0.46 to 0.36)	–0.46 (–1.16 to 0.25)	–0.11 (–0.22 to 0.01)	–0.25 (–0.40 to 0.10)
Difference (% change)	–2.1	–18.5	–4.8	–6.3
*P* value	.66	.11	.04^e^	.12
Levofloxacin				
Anticipated monthly mean^b^	2.19	1.28	1.06	1.52
Observed monthly mean^c^	2.09	1.14	1.17	1.27
Difference (95% CI)^d^	–0.10 (–0.88 to 0.68)	–0.15 (–0.47 to 0.17)	0.11 (–0.00 to 0.24)	–0.24 (–0.81 to 0.33)
Difference (% change)	4.6	–10.9	10.4	–16.4
*P* value	.64	.19	.05	.21

^a^ Data are for 9 of the 10 most commonly prescribed outpatient antibiotics in the US, excluding sulfamethoxazole-trimethoprim (for which complete 2020 data were not available). Agents are listed in rank-order of number of prescriptions filled in quarter 1 2020. Data are presented as number of prescription fills per 1000 persons (based on US Census estimates).

^b^ Volume of prescription fills was anticipated based on prescription fill volumes during each quarter from 2015 through 2019.

^c^ Volume of prescription fills during each quarter of study period (January 1, 2020, to December 31, 2020).

^d^ Volume of prescription fills during the study period was compared with anticipated prescription fills.

^e^ *P* < .05.

Mean monthly cephalexin fills were 10.1% lower than anticipated in quarter 4 (*P* = .05); those of clindamycin were 4.8% lower than anticipated in quarter 3 (*P* = .04). Mean monthly ciprofloxacin fills were 12.2% and 14.3% higher than anticipated in quarters 3 and 4, respectively (*P* = .001 and *P* = .04). Nitrofurantoin and levofloxacin fills were not significantly different from projections in quarter 2, 3, and 4.

## Discussion

This study is the first report of the impact of COVID-19 on US outpatient antibiotic prescriptions throughout 2020. The data tell a mixed story. In April, significant reductions in mean monthly fills of the 4 most commonly prescribed outpatient agents (ie, amoxicillin, azithromycin, amoxicillin-clavulanate, doxycycline) persisted throughout 2020, compared to estimations based on prepandemic trends. However, significantly higher-than-anticipated mean monthly ciprofloxacin fills in quarter 3 and quarter 4 reversed a 5-year trend of consistent reductions in outpatient ciprofloxacin use.^2,3^ For other outpatient antibiotics, previously reported decreases in seasonally adjusted prescriptions in April 2020 were not maintained over the remainder of the year.^1^

Reasons for prescription patterns are unclear. The US Centers for Disease Control estimated that 39% to 41% of adults delayed or deferred medical care in quarter 2, 32% to 41% in quarter 3, and 31% to 35% in quarter 4.^4^ Amoxicillin, azithromycin, amoxicillin-clavulanate, and doxycycline are often used to manage respiratory tract infections.^5^ It is possible that widespread masking and other COVID-19 preventive measures resulted in fewer bacterial respiratory infections, decreased incidence of influenza or other viral infections led to less inappropriate antibiotic use, and clinicians appreciated that SARS-CoV-2 and other viral infections did not require antibiotic treatment.^6^ Lack of sustained reductions in cephalexin and clindamycin fills may reflect primary use against urinary tract and skin or soft tissue infections, respectively. Past reductions in ciprofloxacin use were primarily because of changes in managing urinary tract infections rather than respiratory syndromes.^3^

Patient-level data and information on *International Classification of Diseases, Ninth Revision* codes, and unfilled prescriptions were unavailable, which limited our ability to identify factors associated with prescription fills. Agents included here accounted for over 80% of outpatient antibiotic fills captured by IQVIA; patterns may differ for other agents. We cannot exclude an association of factors unrelated to COVID-19, such as greater attention to antibiotic stewardship. Further studies of antibiotic consumption during the COVID-19 pandemic are needed, with particular attention to drivers and appropriateness of use, regional differences, and antibiotic resistance.
